# Anti-centromere protein B antibody positivity in primary Sjögren’s disease: clinical features and prognostic implications

**DOI:** 10.3389/fimmu.2026.1665077

**Published:** 2026-04-23

**Authors:** Chunxin Lei, Yan Zhang, Jiaqi Chen, Xiya Zhang, Zihan Liu, Xuanyi Zhou, Bojie Tang, Jing Luo, Weijiang Song, Qingwen Tao

**Affiliations:** 1 Graduate School, Beijing University of Chinese Medicine, Beijing, China; 2 National Center for Integrative Medicine, Department of Traditional Chinese Medicine Rheumatism, China-Japan Friendship Hospital, Beijing, China; 3 Traditional Chinese Medicine Department, Peking University Third Hospital, Beijing, China

**Keywords:** anti-centromere protein B, clinical features, prognosis, real-world cohort study, Sjögren’s disease

## Abstract

**Objective:**

To investigate the clinical and immunological features of primary Sjögren’s disease (pSjD) patients with anti-centromere protein B (CENP-B) antibody positivity and to evaluate its prognostic significance.

**Methods:**

This ambispective cohort study included 1,222 patients with pSjD from the China–Japan Friendship Hospital between February 2014 and February 2023, with follow-up through February 2024. Patients were categorized into anti-CENP-B positive and negative groups based on serum testing. Clinical characteristics, immunological features, and outcomes were compared between groups. A subgroup analysis compared patients with isolated CENP-B positivity to those with additional autoantibodies. Statistical analyses were conducted using SPSS 26.0 and R 4.2.3, including descriptive statistics, univariate tests, and Kaplan-Meier survival analysis.

**Results:**

In this study, 100 patients (8.2%) were positive for anti-CENP-B antibody, while 1,122 patients (91.8%) were negative. Compared with the negative group, positive patients were older, more often female, and have higher rates of xerostomia and Raynaud’s phenomenon, but lower frequency of dyspnea. They showed lower platelet counts, higher aspartate aminotransferase, gamma glutamyl transferase, lactate dehydrogenase, total bilirubin, and lower estimated glomerular filtration rate, with reduced frequencies of elevated Immunoglobulin (Ig) G, but increased IgM levels and slightly lower EULAR Sjögren’s Syndrome Disease Activity Index scores. Among anti-CENP-B positive patients, those with isolated anti-CENP-B positivity had milder immunologic abnormalities than those with concomitant autoantibodies. Among patients with available LSGB data, focal lymphocytic infiltration counts were higher in the anti-CENP-B-positive group. Anti-CENP-B positivity was not significantly associated with mortality, cancer, or interstitial lung disease.

**Conclusion:**

Anti-CENP-B positivity in pSjD may identify a different baseline serological and clinical profile, characterized by relatively milder immunologic abnormalities and lower disease activity. However, its prognostic and clinical significance remains uncertain and requires further validation.

## 1 Introduction

Primary Sjögren’s disease (pSjD) is a chronic, systemic autoimmune disease primarily characterized by lymphocytic infiltration of exocrine glands, leading to hallmark symptoms of xerostomia and xerophthalmia. Although initially insidious in onset, the disease can progress to involve multiple organ systems, including the pulmonary, renal, hematologic, and neurologic systems in a substantial proportion of patients during the disease course (1, 2). The current classification criteria, established by the 2016 American College of Rheumatology (ACR) and the European League Against Rheumatism (EULAR), in which anti-SSA/Ro60 antibody positivity plays a pivotal diagnostic role (3). In addition to anti-SSA antibodies, pSjD patients frequently exhibit a broad spectrum of other autoantibodies, including rheumatoid factor (RF), anti-cyclic citrullinated peptide (anti-CCP), anti-Ku, anti-Sm, anti-RNP, and anti-centromere protein B (CENP-B) antibodies. Emerging evidence suggests that the presence of specific autoantibodies may delineate clinically and immunologically distinct subgroups within pSjD. For example, anti-CCP positivity has been associated with increased rates of arthritis, pulmonary involvement, and a higher likelihood of overlapping features with rheumatoid arthritis (4).

Among these, anti-CENP-B antibodies is a major component of anti-centromere antibodies (ACAs). It is traditionally regarded as serological hallmarks of systemic sclerosis (SSc) and primary biliary cholangitis (PBC), and have been linked to Raynaud’s phenomenon and digital sclerosis (5–7). However, recent studies have identified CENP-B antibody positivity in approximately 8.2% to 26% of pSjD patients (8–10), raising the possibility that anti-CENP-B antibody may define a unique immunologic and clinical phenotype within the pSjD spectrum (7, 11, 12). Despite these initial findings, large scale studies characterizing the clinical and serological features of CENP-B positive pSjD patients, particularly those with long-term follow-up data, remain limited. To address this gap, we conducted a comprehensive real-world cohort study to evaluate the clinical manifestations, laboratory parameters, and immunological profiles of pSjD patients positive for anti-CENP-B antibody. We further compared these characteristics with those of CENP-B negative patients, placing special emphasis on major clinical outcomes, including all-cause mortality, cancer, and interstitial lung disease (ILD). This study aims to deepen the understanding of disease heterogeneity in pSjD and to provide evidence supporting refined subtype classification and individualized management strategies.

## 2 Methods

### 2.1 Patient selection

This ambispective cohort study included 1,222 patients with pSjD from the China-Japan Friendship Hospital between February 2014 and February 2023. The study protocol was designed and conducted in accordance with the Declaration of Helsinki and was approved by Ethics Committee of China-Japan Friendship Hospital (Ethical Approval Number: 2024-KY-173). Informed consent was waived for the retrospective part of the study as no personally identifiable information was collected. For the prospective follow-up, written informed consent was obtained from all participants.

### 2.2 Inclusion and exclusion criteria

The inclusion criteria were as follows: (1) fulfillment of the 2016 ACR/EULAR classification criteria for pSjD (3); (2) age between 18 and 75 years. The exclusion criteria were: (1) comorbidities with other connective tissue diseases, such as rheumatoid arthritis, systemic lupus erythematosus, systemic sclerosis; (2) comorbidities with malignant tumours, chronic or active infections; (3) suffering from severe primary cardiovascular, cerebrovascular, hepatic, renal, or endocrine system diseases; (4) substantial missing data in baseline characteristics, key laboratory parameters, or follow-up outcomes that precluded reliable analysis.

### 2.3 Study design

Clinical data for this study cohort were collected retrospectively. Patients were initially stratified into two groups, anti-CENP-B positive and anti-CENP-B negative, based on serum anti-CENP-B antibody results at the time of enrollment. To further characterize the clinical phenotype of anti-CENP-B positive patients, the positive group was subdivided into two subgroups: an isolated positivity group, defined as patients positive for anti-CENP-B antibodies alone, and a concomitant autoantibodies group, defined as patients who were simultaneously positive for anti-CENP-B and other autoantibodies. Given that antinuclear antibody (ANA) is a non-specific screening marker and frequently coexists with anti-CENP-B antibodies due to shared nuclear targets, ANA positivity was permitted in the isolated positivity group.

All patients were followed longitudinally at 6-month intervals through telephone interviews or outpatient visits. Patients were enrolled between February 2014 and February 2023, and follow-up continued until the occurrence of a predefined outcome event—death, cancer, or ILD, or until the predefined cut-off date of February 1, 2024, whichever occurred first. Follow-up time was calculated from the index date to the occurrence of outcomes or last follow-up, and the index date was defined as the date of enrollment at our institution.

### 2.4 Data collection

Clinical data were primarily extracted from the electronic medical record system of the China-Japan Friendship Hospital. Baseline information from patients’ first clinical visit was retrospectively reviewed and documented. Subjective sicca symptoms, including xerostomia and xerophthalmia, were recorded according to the documentation at the first visit. To ensure data accuracy and integrity, a structured database was established using a double-entry system by two independent researchers, with subsequent validation by a third party.

#### 2.4.1 General information and clinical manifestations

1. Demographic characteristics: Information collected included sex, age at the time of consultation, age at disease onset, and disease duration, which was defined as the interval from the initial occurrence of sicca symptoms to the time of presentation. Because formal diagnosis of pSjD is often delayed in routine practice, symptom onset was used as a reference point. However, this variable should be interpreted cautiously.
2. Clinical manifestation: These included symptoms related to pSjD, such as xerostomia, xerophthalmia, fever, fatigue, Raynaud’s phenomenon, purpuric rash, cough, dyspnea, lymphadenopathy, arthritis, rampant caries, and parotid gland enlargement. Clinical manifestations were identified based on physician-documented medical records at cohort entry. Xerostomia and xerophthalmia were defined based on physician-documented patient-reported oral and ocular dryness at the baseline visit. Fever, fatigue, cough, and dyspnea were recorded if present at cohort entry according to the medical record. Raynaud’s phenomenon was defined as recurrent episodic digital color changes triggered by cold exposure or emotional stress. Purpuric rash was defined as non-blanching purpuric skin lesions documented in the medical record. Lymphadenopathy was defined as clinically or radiologically documented lymph node enlargement. Arthritis was defined as physician-documented peripheral joint swelling and/or tenderness considered compatible with inflammatory arthritis. Rampant caries was defined as multiple active dental carious lesions documented by medical history or dental evaluation. Parotid gland enlargement was defined as clinically documented or imaging-confirmed enlargement of the parotid glands.

#### 2.4.2 Assessment of major organ involvement and disease activity

1. Interstitial Lung Disease: The diagnosis of pSjD-associated ILD was evaluated by two experienced radiologist by high-resolution computed tomography of the chest.
2. Splenomegaly: The diagnosis of splenomegaly was based on abdominal ultrasonography. It was defined as a splenic thickness greater than 4 cm, a maximal length exceeding 11 cm, or the presence of a palpable spleen below the left costal margin.
3. Disease Activity: Disease activity was assessed using the EULAR Sjögren’s Syndrome Disease Activity Index (ESSDAI) (13).

#### 2.4.3 Laboratory tests

All laboratory tests were performed using commercial techniques standardized in China-Japan Friendship Hospital. The following baseline indicators were collected:

1. Routine hematological and biochemical parameters: White blood cell (WBC) count, neutrophil count (NEUT), lymphocyte count (LYMPH), red blood cell (RBC) count, hemoglobin (Hb), and platelet (PLT) count; alanine aminotransferase (ALT), aspartate aminotransferase (AST), total bilirubin (TBIL), albumin (ALB), gamma-glutamyl transferase (GGT), lactate dehydrogenase (LDH), creatine kinase (CK), blood urea nitrogen (BUN), serum creatinine (Cr), D-dimer, and estimated glomerular filtration rate (eGFR).
2. Autoantibodies: RF was measured by immunoturbidimetric assay. Anti-cardiolipin (anti-ACL) and anti-β2-glycoprotein I (anti-β2GPI) antibodies were detected using commercial enzyme-linked immunosorbent assay (ELISA) kits. Anti-SSA and all other autoantibodies were assessed using commercial immunoblot kits.
3. Immunological and Inflammatory Markers: Based on the reference ranges of the hospital laboratory, the following indicators were recorded as binary variables, elevated immunoglobulin (Ig) G > 16.2 g/L, elevated IgA > 3.78 g/L, elevated IgM > 2.63 g/L, reduced complement 3 (C3) < 0.70 g/L, reduced complement 4 (C4) < 0.16 g/L, elevated C-reactive protein (CRP) > 8 mg/L, elevated erythrocyte sedimentation rate (ESR) > 20 mm/h. Hypergammaglobulinemia was defined as IgA > 3.78 g/L, IgG > 16.2 g/L, or IgM > 2.63 g/L. Hypocomplementaemia was defined as C3 < 0.7 g/L, or low C4 < 0.16 g/L.
4. Labial salivary gland biopsy (LSGB): When available, LSGB data were collected, including biopsy positivity and focal lymphocytic infiltration counts.

### 2.5 Statistical analysis

All statistical analyses were performed using SPSS version 26.0 and R version 4.2.3. The Shapiro–Wilk test was used to assess the normality of continuous variables. Categorical variables are presented as frequencies and percentages (*%*), and compared between groups using the *χ²* test or Fisher’s exact test, as appropriate. Continuous variables following a normal distribution were expressed as the mean ± standard deviation (*SD*) and compared with the independent samples t test. Non-normally distributed variables were presented as the median with interquartile range (*IQR*) and compared using the Mann–Whitney U test. To account for multiple comparisons, false discovery rate (FDR) correction was performed using the Benjamini–Hochberg procedure.

Survival outcomes, including all-cause mortality, cancer, and ILD, were estimated via Kaplan–Meier curves, and compared via the log-rank test. Given the exploratory nature of this analysis and the absence of *a priori* hypotheses regarding independent prognostic factors, multivariable Cox proportional hazards regression was not pre-specified and was therefore not performed. A two-sided *P* value < 0.05 were considered statistically significant.

Patients with missing essential diagnostic or baseline data were excluded during cohort selection. No imputation was performed in the present study. Analyses were based on complete-case data, except for LSGB-related analyses, which were performed in patients with available biopsy data only.

## 3 Results

**Figure 1:**
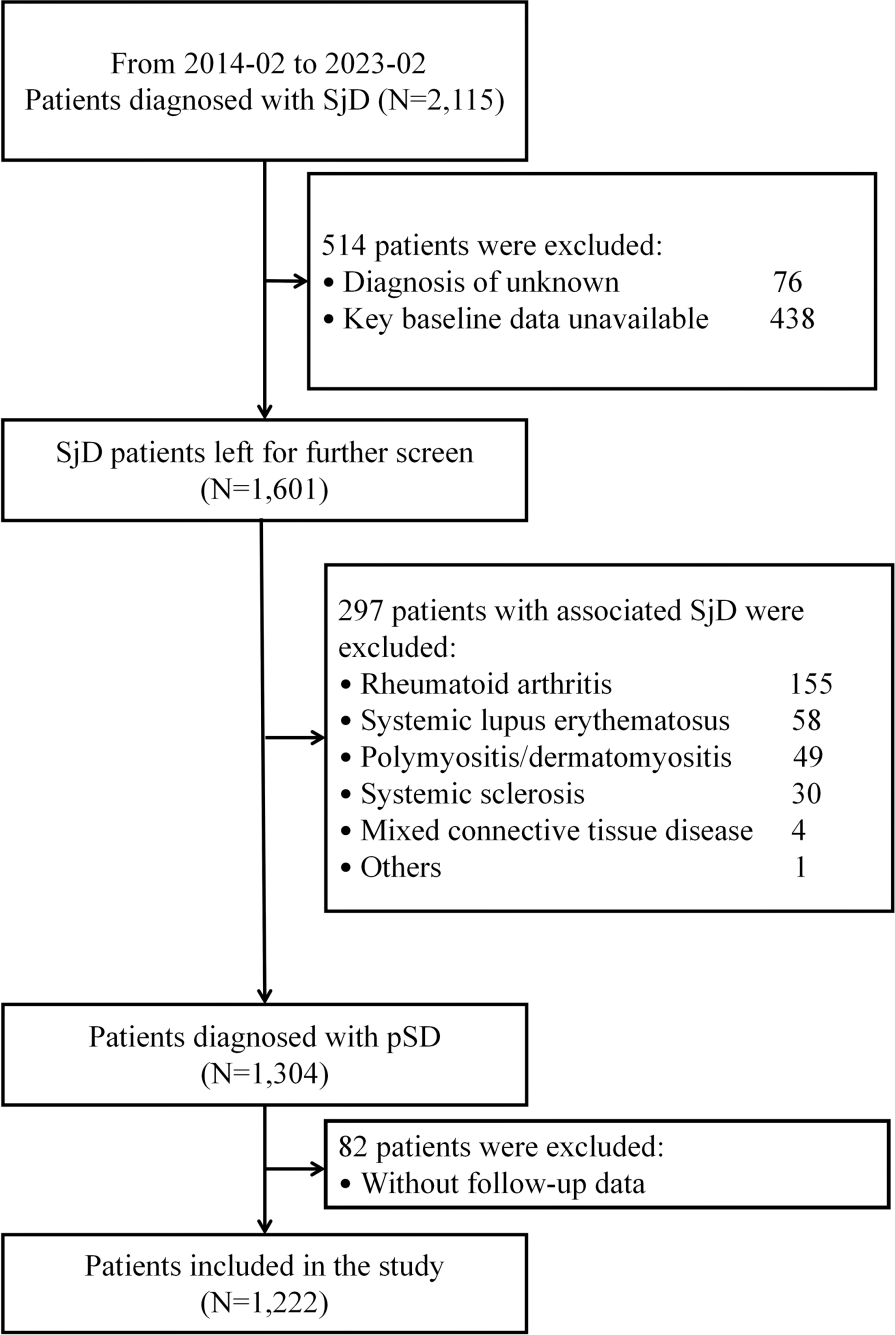
Flowchart for patient selection. SjD, Sjögren’s disease; pSD, primary Sjögren’s disease.

A total of 2,115 patients diagnosed with Sjögren’s disease (SjD) at the China-Japan Friendship Hospital between February 2014 and February 2023 were initially screened. After excluding 811 patients, 1,304 patients with pSD were enrolled for follow-up, 82 of whom were lost to follow-up. Ultimately, 1,222 patients with pSD were included in the final analysis (**Figure 1**).

### 3.1 Comparison of clinical characteristics between anti-CENP-B positive and negative patients with pSjD

In the present study cohort, the prevalence of anti-CENP-B antibody positivity was 8.2%. Compared with anti-CENP-B negative patients, anti-CENP-B positive patients were significantly more likely to be female (95.0% vs. 85.1%, *P* = 0.006), older in age (63.0 [53.5, 69.5] vs. 58.0 [49.0, 67.0] years, *P* = 0.004), and had a higher age at disease onset (55.5 [46.0, 65.0] vs. 53.0 [42.0, 62.0] years, *P* = 0.032). Although xerostomia and xerophthalmia were common in both groups, the anti-CENP-B positive group showed a higher frequency of xerostomia (93.0% vs. 82.7%, *P* = 0.008) and xerophthalmia (85.0% vs. 75.9%, *P* = 0.040). Notably, the incidences of Raynaud’s phenomenon (18.0% vs. 5.1%, *P* < 0.001), rampant caries (37.0% vs. 27.2%, *P* = 0.036) and splenomegaly (9.0% vs. 4.4%, *P* = 0.047) were significantly higher in the anti-CENP-B positive group. In contrast, cough (24.0% vs. 34.9%, *P* = 0.027) and dyspnea (16.0% vs. 31.0%, *P* = 0.002) were significantly less frequent in this group, although the prevalence of ILD did not differ significantly between the two groups (33.0% vs. 41.2%, *P* = 0.110). No significant differences were observed between in fever, fatigue, lymphadenopathy, arthritis, or Parotid enlargement (**Table 1**). After FDR adjustment, female sex, older age, xerostomia, Raynaud’s phenomenon, and dyspnea remained statistically significant, whereas age at onset, xerophthalmia, rampant caries, splenomegaly, and cough were no longer statistically significant (**Supplementary Table 1**).

**Table 1:** Comparison of the clinical features of the anti-CENP-B antibody positive and negative pSjD patients.

Characteristics	Anti-CENP-B antibody positivity, n=100	Anti-CENP-B antibody negative, n=1122	*χ2*	*P*
Gender (Female)	95(95.0)	955(85.1)	——	0.006
Age (years)	63.0(53.5, 69.5)	58.0(49.0, 67.0)	——	0.004
Age at onset (years)	55.5(46.0, 65.0)	53.0(42.0, 62.0)	——	0.032
Disease duration (months)	48.0(12.0, 93.0)	36.0(8.0, 96.0)	——	0.188
Xerostomia	93(93.0)	928(82.7)	7.075	0.008
Xerophthalmia	85(85.0)	852(75.9)	4.218	0.040
Fever	9(9.0)	139(12.4)	0.99	0.320
Raynaud's phenomenon	18(18.0)	57(5.1)	26.604	<0.001
Rampant caries	37(37.0)	305(27.2)	4.390	0.036
Purpuric rash	5(5.0)	84(7.5)	0.841	0.359
Fatigue	50(50.0)	519(46.3)	0.517	0.472
Cough	24(24.0)	392(34.9)	4.892	0.027
Dyspnea	16(16.0)	348(31.0)	9.899	0.002
Lymphadenopathy	21(21.0)	197(17.6)	0.742	0.389
Arthritis	11(11.0)	91(8.1)	1.002	0.317
Parotid enlargement	7(7.0)	78(7.0)	0.000	0.986
Splenomegaly	9(9.0)	49(4.4)	——	0.047
Interstitial lung disease	33(33.0)	462(41.2)	2.547	0.110

### 3.2 Comparison of laboratory parameters between anti-CENP-B positive and negative pSjD patients

A total of 100 anti-CENP-B positive patients and 1,122 anti-CENP-B negative patients were included in the laboratory analysis. Compared with the anti-CENP-B negative group, the anti- CENP-B positive group had a lower platelet counts (173.0 [139.25, 211.50] vs. 199.0 [155.00, 245.00] ×10^9^/L, *P* < 0.001). No significant differences were observed between the two groups in WBC, NEUT, LYMPH, RBC, or Hb levels (*P* > 0.05 for all). Meanwhile, the anti-CENP-B positive group had higher levels of AST (23.00 [19.00, 30.75] vs. 21.00 [17.00, 26.00] IU/L, *P* = 0.003), GGT (27.00 [17.00, 57.50] vs. 22.00 [15.00, 36.00] IU/L, *P* = 0.002), TBIL (11.02 [8.52, 14.83] vs. 9.95 [7.32, 13.42] μmol/L, *P* = 0.009), CK (55.50 [41.00, 86.75] vs. 51.00 [36.00, 74.00] IU/L, *P* = 0.025), LDH (188.00 [158.00, 227.75] vs. 177.00 [152.00, 218.00] IU/L, *P* = 0.033), and NA (142.50 [139.00, 143.00] vs. 140.00 [138.00, 141.00] mmol/L, *P* = 0.002). Whereas eGFR was lower in the anti-CENP-B positive group compared to the negative group (91.49 [80.86, 101.52] vs. 97.02 [85.15, 105.61] mL/min/1.73m², *P* = 0.001). No significant differences were found in ALT, ALB, BUN, Cr, K, Cl, or D-dimer (all *P* > 0.05) (**Table 2**). After FDR adjustment, PLT, AST, TBIL, GGT, NA, and eGFR remained statistically significant, whereas CK and LDH were no longer statistically significant (**Supplementary Table 1**).

**Table 2:** Comparison of laboratory parameters between CENP-B positive and negative pSjD patients.

Laboratory parameters	Anti-CENP-B antibody positivity, n=100	Anti-CENP-B antibody negative, n=1122	*P*
WBC, ×10^9^/L	4.78(3.83, 6.03)	5.09(3.88, 6.66)	0.108
NEUT, ×10^9^/L	2.74(2.03, 3.70)	2.97(2.10, 4.27)	0.131
LYMPH, ×10^9^/L	1.39(1.12, 1.80)	1.46(1.07, 1.87)	0.694
RBC, ×10^9^/L	3.98(3.64, 4.33)	4.09(3.73, 4.41)	0.099
Hb, g/L	122.50(111.25, 132.75)	124.00(112.00, 134.00)	0.284
PLT, ×10^9^/L	173(139.25, 211.50)	199.00(155.00, 245.00)	<0.001
ALT, IU/L	20.00(14.00, 33.00)	19.00(13.00, 28.00)	0.262
AST, IU/L	23.00(19.00, 30.75)	21.00(17.00, 26.00)	0.003
TBIL, μmol/L	11.02(8.52, 14.83)	9.95(7.32, 13.42)	0.009
ALB, g/L	40.00(38.00, 42.93)	40.00(37.20, 42.33)	0.331
GGT, IU/L	27.00(17.00, 57.50)	22.00(15.00, 36.00)	0.002
LDH, IU/L	188.00(158.00, 227.75)	177.00(152.00, 218.00)	0.033
CK, IU/L	55.50(41.00, 86.75)	51.00(36.00, 74.00)	0.025
BUN, mmol/L	4.76(3.82, 5.96)	4.66(3.72, 5.75)	0.303
Cr, μmol/L	60.60(53.03, 68.83)	59.20(51.70, 69.70)	0.476
K, mmol/L	4.00(3.80, 4.20)	3.90(3.70, 4.13)	0.285
NA, mmol/L	140.50(139.00, 143.00)	140.00(138.00, 141.00)	0.002
Cl, mmol/L	106.00(105.00, 108.00)	106.00(104.00, 108.00)	0.055
eGFR, ml/min/1.73m^2^	91.49(80.86, 101.52)	97.02(85.15, 105.61)	0.001
D-Dimer, mg/L	0.48(0.28, 0.75)	0.42(0.28, 0.86)	0.746

WBC, white blood cell; NEUT, neutrophil; LYMPH, lymphocyte; RBC, red blood cell; Hb, hemoglobin; PLT, platelet; ALT, alanine transaminase; AST, aspartate aminotransferase; TBIL, total bilirubin; ALB, albumin; GGT, gamma glutamyl transferase; LDH, lactate dehydrogenase; CK, creatine kinase; BUN, blood urea nitrogen; Cr, serum creatinine; eGFR, estimated glomerular filtration rate.

### 3.3 Comparison of immunological features and ESSDAI scores between anti-CENP-B positive and negative pSjD patients

Significant differences in immunological features were observed between anti-CENP-B positive patients (n = 100) and anti-CENP-B negative patients (n = 1,122) (**Table 3**). Compared with the anti-CENP-B negative group, the anti-CENP-B positive group had a lower frequencies of hypergammaglobulinemia (47.0% vs. 57.5%, *P* = 0.043), elevated IgG (31.0% vs. 47.6%, *P* = 0.001), elevated CRP (15.0% vs. 26.6%, *P* = 0.011), and elevated ESR (41.0% vs. 52.6%, *P* = 0.026), whereas the frequency of elevated IgM was higher (13.0% vs. 6.9%, *P* = 0.024). In addition, the anti-CENP-B positive group showed lower levels of IgG (13.95 [11.43, 17.50] vs. 15.90 [12.60, 20.40] g/L, *P* = 0.003), C3 (0.78 [0.69, 0.92] vs. 0.84 [0.72, 0.98] g/L, *P* = 0.017), and ESR (14.00 [8.25, 33.00] vs. 23.00 [11.00, 43.00] mm/h, *P* = 0.012), as well as lower ESSDAI scores (6.00 [3.00, 14.00] vs. 7.00 [3.00, 14.00], P = 0.009). No significant differences were observed in the prevalence of hypocomplementaemia or elevated IgA, or in the levels of C4, IgA, IgM, or CRP (all *P* > 0.05).

**Table 3:** Comparison of immunological features and disease activity between CENP-B positive and negative pSjD patients.

Immunological features and disease activity	Anti-CENP-B antibody positivity, n=100	Anti-CENP-B antibody negative, n=1122	*χ2*	*P*
Hypergammaglobulinemia	47(47.0)	645(57.5)	4.111	0.043
IgG, g/L	13.95(11.43, 17.50)	15.90(12.60, 20.40)	——	0.003
Hyper-IgG (> 16.2 g/L)	31(31.0)	534(47.6)	10.170	0.001
IgA, g/L	2.89(1.87, 3.74)	2.89(2.02, 3.83)	——	0.426
Hyper-IgA (> 3.78 g/L)	23(23.0)	292(26.0)	0.439	0.508
IgM, g/L	1.25(0.79, 1.79)	1.04(0.70, 1.52)	——	0.408
Hyper-IgM (> 2.63 g/L)	13(13.0)	77(6.9)	5.069	0.024
Hypocomplementemic	48(48.0)	464(41.4)	1.666	0.197
C3, g/L	0.78(0.69, 0.92)	0.84(0.72, 0.98)	——	0.017
C4, g/L	0.18(0.14, 0.21)	0.18(0.14, 0.23)	——	0.154
CRP, mg/L	0.28(0.17, 0.56)	0.31(0.19, 0.80)	——	0.082
Elevated CRP (> 8 mg/L)	15(15.0)	298(26.6)	6.439	0.011
ESR, mm/h	14.00(8.25, 33.00)	23.00(11.00, 43.00)	——	0.012
Elevated ESR (> 20 mm/h)	41(41.0)	590(52.6)	4.934	0.026
ESSDAI scores	6.00(3.00, 14.00)	7.00(3.00, 14.00)	——	0.009
Positive anti-ANA	100(100.0)	1102(98.2)	——	0.400
ANA titers ≥1:320	41(41.0)	321(28.6)	6.761	0.009
Positive RF	36(36.0)	485(43.2)	1.960	0.161
Positive anti-AMA-M2	14(14.0)	89(7.9)	4.380	0.036
Positive anti-PM-Scl	3(3.0)	8(0.7)	——	0.054
Positive anti-ACL	6(6.0)	74(6.6)	0.053	0.818
Positive anti-β2GP1	5(5.0)	55(4.9)	——	1.000
Positive anti-dsDNA	8(8.0)	95(8.5)	0.026	0.872
Centriole	1(1.0)	30(2.7)	——	0.507
Positive anti-Sm	0(0.0)	10(0.9)	——	1.000
Positive anti-SSA	50(50.0)	814(72.5)	22.538	<0.001
Positive anti-Ro-52	53(53.0)	646(57.6)	0.785	0.376
Positive anti-SSB	14(14.0)	329(29.3)	10.677	0.001
Positive anti-RNP	6(6.0)	82(7.3)	0.235	0.628
Positive anti-Scl-70	3(3.0)	10(0.9)	——	0.083
Positive LSGB	62/63(98.4)	524/564(92.9)	——	0.109
Focal lymphocytic infiltration count	3(2,5)	2(1,4)	——	<0.001

Ig, immunoglobulin; C3, complement 3; C4 complement 4; RF, rheumatoid factor; ESR, erythrocyte sedimentation rate; CRP, C-reactive protein; ANA, antinuclear antibodies; ESSDAI, EULAR Sjögren’s syndrome disease activity index; AMA-M2, anti-mitochondrial antibody M2; ACL, anti-cardiolipin; LSGB, Labial salivary gland biopsy.

In terms of autoantibodies, patients in the anti-CENP-B positive group had a higher proportion of ANA titers ≥ 1:320 (41.0% vs. 28.6%, *P* = 0.009) and higher positivity for anti-AMA-M2 (14.0% vs. 7.9%, *P* = 0.036), whereas the positivity rates for anti-SSA (50.0% vs. 72.5%, *P* < 0.001) and anti-SSB (14.0% vs. 29.3%, *P* = 0.001) were lower. No significant differences were observed in RF, anti-Sm, anti-RNP, anti-Scl-70, anti-ACL, anti-β2GP1, anti-PM-Scl, anti-dsDNA, anti-Ro-52, or Centriole positivity (all *P* > 0.05).

LSGB data were available for 634 of the 1,222 included patients (51.9%). Among them, 586 were biopsy-positive, 41 were biopsy-negative, and 7 had missing pathological interpretation. Among patients with available biopsy, the proportion of biopsy positivity did not differ significantly between the two groups [62/63 (98.4%) vs. 524/564 (92.9%), *P* = 0.109], whereas the focal lymphoid cell infiltration count was higher in anti-CENP-B positive group [3 (2, 5) vs. 2 (1, 4), *P* < 0.001].

After FDR adjustment, IgG, elevated IgG, elevated CRP, ESR, ESSDAI score, ANA titers ≥ 1:320, anti-SSA, anti-SSB, and focal lymphocytic infiltration count remained statistically significant, whereas hypergammaglobulinemia, elevated IgM, C3, elevated ESR, and anti-AMA-M2 were no longer statistically significant (**Supplementary Table 1**).

### 3.4 Comparison of clinical and laboratory features between pSjD patients with isolated anti-CENP-B positivity and those with concomitant autoantibodies

To further characterize anti-CENP-B positive patients, the anti-CENP-B positive group was subdivided into an isolated anti-CENP-B positive group (n = 20) and a concomitant autoantibody positivity group (n = 80). No significant differences in baseline demographic or clinical characteristics were observed between the two subgroups (all *P* > 0.05). Further comparisons of serological and laboratory parameters showed that, compared with the isolated anti-CENP-B positive group, the concomitant autoantibodies positive group had lower levels of Hb (121.00 [108.00, 130.75] vs. 130.00 [119.50, 135.50] g/L, *P* = 0.015), CK (53.50 [40.25, 81.75] vs. 88.50 [45.25, 103.50] IU/L, *P* = 0.021), and C4 (0.17 [0.14, 0.20] vs. 0.19 [0.17, 0.22] g/L, *P* = 0.020). In addition, the concomitant autoantibody positivity group had higher frequencies of hypergammaglobulinemia (53.8% vs. 20.0%, *P* = 0.007), elevated IgG (36.3% vs. 10.0%, *P* = 0.023), hypocomplementemia (53.8% vs. 25.0%, *P* = 0.021), low C3 (32.5% vs. 10.0%, *P* = 0.045), and elevated ESR (46.3% vs. 20.0%, *P* = 0.033) (**Table 4**; **Supplementary Table 2**). Among anti-CENP-B positive patients with available biopsy data, biopsy positivity was observed in 20/20 (100.0%) of the isolated anti-CENP-B positive subgroup and 47/48 (97.9%) of the concomitant antibody positive subgroup (*P* = 1.000), whereas the lymphocytic infiltration focus count did not differ significantly between the two subgroups (3 [2, 3.5] vs. 4 [2, 5], *P* = 0.137). Because the isolated anti-CENP-B positive subgroup was small, these subgroup comparisons should be interpreted with caution.

**Table 4:** Comparison of laboratory parameters between anti-CENP-B alone and co-positive subgroups in pSjD patients.

Clinical and laboratory features	Isolated Anti-CENP-B antibody (+), n = 20	Concomitant Anti-CENP-B antibodies (+), n = 80	*χ2*	*OR*(95%CI)	*P*
Hb, g/L	130.00(119.50,135.50)	121.00(108.00,130.75)	——	——	0.015
CK, IU/L	88.50(45.25, 103.50)	53.50(40.25,81.75)	——	——	0.021
Hypergammaglobulinemia	4(20.0)	43(53.8)	7.316	0.215(0.066,0.7)	0.007
Hyper-IgG (> 16.2g/L)	2(10.0)	29(36.3)	5.154	0.195(0.042,0.903)	0.023
Hypocomplementemic	5(25.0)	43(53.8)	5.298	0.287(0.095,0.865)	0.021
Low C3 (<70 g/L)	2(10.0)	26(32.5)	4.018	0.231(0.05,1.07)	0.045
C4, g/L	0.19(0.17,0.22)	0.17(0.14,0.20)	——	——	0.02
ESR, mm/h	10.00(5.50,15.50)	16.00(11.00,36.50)	——	——	0.006
Elevated ESR (> 20 mm/h)	4(20.0)	37(46.3)	4.558	0.291(0.089,0.946)	0.033

C4, complement 4; ESR, erythrocyte sedimentation rate; Hb, hemoglobin; CK, creatine kinase; Ig, immunoglobulin.

Odds ratio (*OR*) with 95% confidence interval (*CI*) are presented for categorical variables only. Continuous variables are presented as median (*IQR*).

### 3.5 Prognostic analysis of CENP-B antibody positivity

During follow-up, a total of 124 deaths, 34 cases of cancer, and 29 incident ILD events were observed. Regarding ILD, 487 patients had ILD at baseline, while the remaining 735 patients were included in the cohort for incident ILD events. Of these, 42 patients were lost to follow-up, leaving 693 patients for analysis. Kaplan-Meier survival analyses showed no statistically significant differences between the anti-CENP-B positive and negative groups in overall survival, incidence of cancer, or ILD (all *P* > 0.05) (**Supplementary Figure 1**). Therefore, no further Cox regression analysis was performed. In addition, by the end of follow-up, 6 patients had been diagnosed with PBC and 2 with autoimmune hepatitis (AIH), all of whom were anti-CENP-B negative, whereas no cases of secondary SSc were observed (**Supplementary Table 3**).

## 4 Discussion

In recent years, an increasing variety of autoantibody subtypes have been identified in patients with pSjD. Among them, anti-CENP-B antibodies, a member of the ACAs family, have been detected in a subset of pSjD patients and have garnered growing interest for their potential clinical and prognostic implications (5). Although anti-CENP-B antibodies are traditionally recognized as serological markers of SSc and PBC, their clinical significance in pSjD remains incompletely understood. In this study, we systematically characterized the clinical, serological, and immunological features of pSjD patients with anti-CENP-B positivity and further conducted subgroup analyses within the anti-CENP-B positive population, while also evaluating their association with major outcome events during follow-up.

In our real-world cohort, the prevalence of anti-CENP-B antibody positivity was 8.2%. This rate is consistent with the prevalence reported in the existing literature, which ranges from 8.2% to 26% (8–10). Compared with anti-CENP-B negative patients, anti-CENP-B positive patient were more often female and older at enrollment. After FDR correction, the most robust clinical differences were a higher frequency of xerostomia, Raynaud’s phenomenon and a lower frequency of dyspnea, whereas xerophthalmia and rampant caries were not. The increased frequency of raynaud’s phenomenon is more consistent with the recognized anticentromere-associated clinical spectrum (14, 15), in which vascular manifestations are more prominent. However, we acknowledge that the absolute prevalence of xerostomia and xerophthalmiaoral in our cohort was lower than that reported in some classical pSjD cohorts. This may be related to the clinical setting and case mix of our study. As China-Japan Friendship Hospital is a national respiratory medicine center, the cohort included both outpatients and hospitalized patients and may therefore have included a relatively higher proportion of patients presenting with systemic manifestations. Importantly, previous studies suggest that, in some patients, systemic features may precede the onset of overt sicca syndrome, defining an “occult” form of pSjD with non-sicca onset (16–19). Therefore, our cohort may not fully represent a typical symptom-enriched pSjD population.

In the laboratory and immunological analyses, anti-CENP-B positivity was associated with lower platelet counts, lower IgG levels and lower frequency of elevated IgG, lower inflammatory activity as reflected by ESR, elevated CRP, and ESSDAI, higher ANA titers, lower anti-SSA and anti-SSB positivity. Indicate that anti-CENP-B-positive patients in this cohort had lower baseline systemic inflammatory and humoral immune activity. Previous studies indicate that different autoantibody patterns in pSjD may identify biologically distinct subsets rather than a single uniform disease process, it shown that anti-Ro52 positive pSjD patients tend to have higher disease activity (20, 21), possibly due to TRIM21/Ro52 overexpression, which suppresses cell proliferation and enhances CD40-mediated apoptosis (22). In our study, the anti-SSA/Ro60 and anti-SSB antibody positivity rates were significantly lower in the anti-CENP-B positive group, and although the difference in anti-Ro52 antibody positivity did not reach statistical significance, a lower trend was observed. Because anti-Ro/SSA and anti-La/SSB antibodies are classically associated with more severe exocrine dysfunction, recurrent parotid enlargement, and stronger minor salivary gland lymphocytic infiltration (14), this serological pattern supports the interpretation that anti-CENP-B positive patients may be less likely to follow the conventional Ro/La-associated pathway of heightened humoral immune activation. Among patients with available LSGB data, the biopsy positivity rate did not differ significantly between anti-CENP-B positive and negative patients, whereas the focal lymphocytic infiltration count was higher in the anti-CENP-B positive group. These findings suggest that anti-CENP-B positive pSjD patients may have a different baseline serological and immunological profile from anti-CENP-B negative patients. In particular, the lower frequency of anti-SSA and anti-SSB positivity was observed in our cohort is consistent with previous research, supporting that this population may represent a distinct serological subtype of pSjD (7, 8). Taken together, our findings suggest that anti-CENP-B positivity in pSjD is more likely to represent a distinct clinical-serological and immunological subset than a directly pathogenic antibody. At present, our data do not establish a direct functional role for anti-CENP-B antibodies in disease biology, and further mechanistic studies are still needed.

Rischmueller et al. (23) proposed that antibody diversity in pSjD is closely linked to HLA class II genotypes, particularly the DR3-DQ2 haplotype, which is associated with a strong anti-Ro/La response and heightened immune activation. In contrast, anti-CENP-B, which is not associated with the Ro/La ribonucleoprotein complex, may arise through distinct immunological pathways, thereby contributing to a different serological profile. Our findings are consistent with this concept, although the present study was not designed to investigate underlying immunogenetic mechanisms. Importantly, our results do not establish a direct pathogenic role for anti-CENP-B antibodies themselves. Rather, anti-CENP-B positivity may be better interpreted as a marker of a particular immunological subset characterized by lower anti-SSA/SSB positivity, lower IgG-related activity, and a different pattern of systemic and glandular manifestations. Whether anti-CENP-B antibodies are merely epiphenomena of this immune background or participate directly in tissue injury remains uncertain and requires dedicated mechanistic studies.

In the present study, anti-CENP-B positive patients also showed statistically higher AST, TBIL, and GGT levels and lower eGFR. However, the absolute differences were modest, and the median values were largely within or near the normal range. Therefore, these findings should not be interpreted as evidence of clinically overt liver or renal involvement. With respect to renal function, both groups remained within a generally preserved eGFR range, and the lower eGFR observed in anti-CENP-B positive patients may partly reflect their older age rather than clinically meaningful renal impairment. Likewise, although previous studies have suggested possible associations between ACA positivity and autoimmune liver disease (9, 23), our data do not support overinterpretation of these biochemical differences as definite evidence of increased liver disease burden in anti-CENP-B positive patients.

The follow-up data further support a cautious interpretation of organ-specific risk. During follow-up, 6 patients developed PBC and 2 developed AIH, all of whom were anti-CENP-B negative, and no cases of secondary SSc were observed. In addition, Kaplan-Meier analyses showed no significant differences between anti-CENP-B positive and anti-CENP-B negative patients in all-cause mortality, cancer incidence, or incident ILD. These findings suggest that anti-CENP-B positivity was not associated with worse major outcome events during the observed follow-up period in this cohort. Nevertheless, these negative results should also be interpreted cautiously because the number of outcome events was limited and follow-up duration may not have been sufficient to detect long-term differences.

Subgroup analysis within the anti-CENP-B positive population showed that patients with concomitant autoantibody positivity had lower hemoglobin, C4 levels and higher ESR, together with higher frequencies of hypergammaglobulinemia, elevated IgG, hypocomplementaemia, low C3, and elevated ESR, compared with patients with isolated anti-CENP-B positivity. These results indicate that the concomitant autoantibody positive subgroup had higher baseline humoral immune and inflammatory activity than the isolated anti-CENP-B positive subgroup. However, due to the small sample size of the isolated anti-CENP-B-positive subgroup, this analysis should be viewed in context.

Anti-CENP-B, which serves as a key autoantibody marker for SSc and PBC, is often linked to specific manifestations, most notably Raynaud’s phenomenon and sclerosis of the finger tips (5–7). It has been suggested that pSjD patients with anti-CENP-B antibody positivity may have different clinical and immunological features from those of SSc (7, 11, 12). Tanaka et al. (12) reported that pSjD patients differed significantly from SSc patients with respect to anti-CENP-C and HP1α antibody profiles, supporting the possibility that ACA-positive pSjD may represent a subgroup distinct from classical SSc-associated ACA positivity. Our findings add to this body of evidence by showing that anti-CENP-B positivity in pSjD is associated with a particular baseline serological profile, especially lower anti-SSA/SSB positivity and lower inflammatory activity. Whether PBC should be classified as a ‘secondary’ condition in the context of pSjD is debatable, because its onset can occur at different time point during the disease course of pSjD (24–27). Salliot et al. (28) showed that in patients with co-morbidity of SjD and SSc, ACAs were frequently detected together with anti-SSA/Ro60 and anti-SSB antibodies, and these patients often comorbid with a third autoimmune disease, especially PBC, showing a tendency toward autoimmune proliferation, supporting its evolution as an overlapping syndrome rather than the subordination of one disease to another. More interestingly, it was found that when SSc was co-morbid with SjD, the severity of SSc itself was instead reduced, with a significantly lower incidence of pulmonary fibrosis in particular, in line with the more benign immunological manifestations seen in patients with CENP-B antibody positive pSjD in the present study. Park Y et al. (29), in a large-sample retrospective cohort of pSjD, found that splenomegaly, high IFI-HEp-2 titer and ACA were independent predictors of liver involvement. In the present study, anti-CENP-B positivity in pSjD is associated with a particular baseline serological profile, especially lower anti-SSA/SSB positivity and lower inflammatory activity. And it may be better regarded as a marker of immune heterogeneity and overlap tendency rather than as a direct indicator of progression to another autoimmune disease.

However, this study also has several limitations. First, the sample size of the isolated anti-CENP-B positive subgroup was small. This subgroup analysis was exploratory in nature and was not prospectively powered for formal between-subgroup comparisons. Therefore, some subgroup differences may not have reached statistical significance, because of limited statistical power, and these findings require confirmation in larger cohorts. Second, the prognostic analyses in this study were based on Kaplan-Meier survival comparisons, and no further Cox regression analysis was performed because no significant between-group differences were observed. Thus, the absence of statistically significant survival differences should be interpreted cautiously, as it may have been influenced by limited sample size, a small number of outcome events, and insufficient follow-up duration. Third, although LSGB data were available in a substantial subset of patients, histopathological evaluation was not available for the entire cohort, which may have introduced selection bias. Objective measures of salivary gland dysfunction were not systematically available and therefore could not be included in the analysis. Finally, as a single-center study conducted in a relatively homogeneous population, the generalizability of our findings is limited. Future multicenter, prospective, and multiethnic studies are warranted to validate these findings and to further clarify the clinical significance of anti-CENP-B positivity in the pSjD spectrum.

In conclusion, anti-CENP-B positivity in pSjD was associated with a distinct baseline serological and immunological profile in this cohort, characterized by lower anti-SSA/SSB positivity, lower inflammatory and IgG-related activity, and higher focal lymphocytic infiltration in the subgroup with available biopsy data. At the same time, anti-CENP-B positivity was not associated with poorer major outcome events during follow-up. Therefore, anti-CENP-B is more likely to represent a marker of immune heterogeneity within pSjD than a direct indicator of disease progression.

## Data Availability

The raw data supporting the conclusions of this article are available from the corresponding author upon reasonable request, without undue restriction.
