# Supplementary material for: Anti-centromere protein B antibody positivity in primary Sjögren’s disease: clinical features and prognostic implications
Source: Front Immunol. 2026 Apr 23;17:1665077. doi: 10.3389/fimmu.2026.1665077 (PMC13149392; doi:10.3389/fimmu.2026.1665077)
Supplement: Supplementary file 1 [file Table1.docx]

Supplementary Material

# 1 Supplementary Tables

**Supplementary Table 1. | Raw and FDR-adjusted P values for comparisons of clinical, laboratory, and immunological variables between anti-CENP-B-positive and anti-CENP-B-negative patients with primary Sjögren’s disease**

| **Supplementary 1. Raw and FDR-adjusted P values for comparisons of clinical, laboratory, and immunological variables between anti-CENP-B-positive and anti-CENP-B-negative patients with primary Sjögren’s disease** | | |
| --- | --- | --- |
| **Variables** | **Raw *P*** | **FDR-adjusted *P*** |
| Gender (Female) | 0.006 | 0.030 |
| Age (years) | 0.004 | 0.022 |
| Age at onset (years) | 0.032 | 0.086 |
| Disease duration (months) | 0.188 | 0.299 |
| Xerostomia | 0.008 | 0.035 |
| Xerophthalmia | 0.04 | 0.093 |
| Fever | 0.32 | 0.439 |
| Raynaud's phenomenon | 0.001 | 0.010 |
| Rampant caries | 0.036 | 0.087 |
| Purpuric rash | 0.359 | 0.474 |
| Fatigue | 0.472 | 0.555 |
| Cough | 0.027 | 0.076 |
| Dyspnea | 0.002 | 0.014 |
| Lymphadenopathy | 0.389 | 0.495 |
| Arthritis | 0.317 | 0.439 |
| Parotid enlargement | 0.986 | 1.000 |
| Splenomegaly | 0.047 | 0.103 |
| Interstitial lung disease | 0.11 | 0.193 |
| WBC, ×10^9^/L | 0.108 | 0.193 |
| NEUT, ×10^9^/L | 0.131 | 0.224 |
| LYMPH, ×10^9^/L | 0.694 | 0.759 |
| RBC, ×10^9^/L | 0.099 | 0.187 |
| Hb, g/L | 0.284 | 0.416 |
| PLT, ×10^9^/L | 0.001 | 0.010 |
| ALT, IU/L | 0.262 | 0.399 |
| AST, IU/L | 0.003 | 0.018 |
| TBIL, μmol/L | 0.009 | 0.035 |
| ALB, g/L | 0.331 | 0.446 |
| GGT, IU/L | 0.002 | 0.014 |
| LDH, IU/L | 0.033 | 0.086 |
| CK, IU/L | 0.025 | 0.076 |
| BUN, mmol/L | 0.303 | 0.433 |
| Cr, μmol/L | 0.476 | 0.555 |
| K, mmol/L | 0.285 | 0.416 |
| NA, mmol/L | 0.002 | 0.014 |
| Cl, mmol/L | 0.055 | 0.113 |
| eGFR, ml/min/1.73m^2^ | 0.001 | 0.010 |
| D-Dimer, mg/L | 0.746 | 0.803 |
| Hypergammaglobulinemia | 0.043 | 0.097 |
| IgG, g/L | 0.003 | 0.018 |
| Hyer-IgG (> 16.2 g/L) | 0.001 | 0.010 |
| IgA, g/L | 0.426 | 0.514 |
| Hyer-IgA (> 3.78 g/L) | 0.508 | 0.574 |
| IgM, g/L | 0.408 | 0.501 |
| Hyer-IgM (> 2.63 g/L) | 0.024 | 0.076 |
| Hypocomplementemic | 0.197 | 0.306 |
| C3, g/L | 0.017 | 0.057 |
| C4, g/L | 0.154 | 0.257 |
| CRP, mg/L | 0.082 | 0.161 |
| Elevated CRP (> 8 mg/L) | 0.011 | 0.041 |
| ESR, mm/h | 0.012 | 0.042 |
| Elevated ESR (> 20 mm/h) | 0.026 | 0.076 |
| ESSDAI scores | 0.009 | 0.035 |
| Positive anti-ANA | 0.4 | 0.500 |
| ANA titres ≥1:320 | 0.009 | 0.035 |
| Positive RF | 0.161 | 0.262 |
| Positive anti-AMA-M2 | 0.036 | 0.087 |
| Positive anti-PM-Scl | 0.054 | 0.113 |
| Positive anti-ACL | 0.818 | 0.868 |
| Positive anti-β2GP1 | 1.000 | 1.000 |
| Positive anti-dsDNA | 0.872 | 0.911 |
| Centriole | 0.507 | 0.574 |
| Positive anti-Sm | 1.000 | 1.000 |
| Positive anti-SSA | 0.001 | 0.010 |
| Positive anti-Ro-52 | 0.376 | 0.487 |
| Positive anti-SSB | 0.001 | 0.010 |
| Positive anti-RNP | 0.628 | 0.698 |
| Positive anti-Scl-70 | 0.083 | 0.161 |
| Labial salivary gland biopsy | 0.109 | 0.193 |
| focal lymphoid cell infiltration | 0.001 | 0.010 |

WBC, white blood cell; NEUT, neutrophil; LYMPH, lymphocyte; RBC, red blood cell; Hb, hemoglobin; PLT, platelet; ALT, alanine transaminase; AST, aspartate aminotransferase; TBIL, total bilirubin; ALB, albumin; GGT, gamma glutamyl transferase; LDH, lactate dehydrogenase; CK, creatine kinase; BUN, blood urea nitrogen; Cr, serum creatinine; eGFR, estimated glomerular filtration rate; Ig, immunoglobulin; C3, complement 3; C4, complement 4; RF, rheumatoid factor; ESR, erythrocyte sedimentation rate; CRP, C-reactive protein; ANA, antinuclear antibodies; ESSDAI, EULAR Sjögren’s syndrome disease activity index; AMA-M2, anti-mitochondrial antibody M2; ACL, anti-cardiolipin; LSGB, Labial salivary gland biopsy.

**Supplementary Table 2. | Differences in clinical features and laboratory tests in the isolated anti-CENP-B positive group compared to the concomitant positive group of primary Sjögren’s disease patients**

| Supplementary Table 2. Differences in clinical features and laboratory tests in the isolated anti-CENP-B positive group compared to the concomitant positive group of primary Sjögren’s disease patients | | | | | |
| --- | --- | --- | --- | --- | --- |
|  | Isolated Anti-CENP-B antibody(+) , n = 20 | Concomitant Anti-CENP-B antibodies(+), n = 80 | *χ2* | *OR* (95%CI) | *P* |
| Gender (Female) | 18(90.0) | 77(96.3) | — | 0.351 (0.055,2.255) | 0.261 |
| Age (years) | 64.65±10.35 | 60.59±11.34 | — | — | 0.216 |
| Age at onset (years) | 59.55±12.40 | 54.11±13.84 | — | — | 0.14 |
| Disease duration (months) | 54.00(12.00,84.00) | 48.00(12.00,114.00) | — | — | 0.51 |
| Xerostomia | 18(90.0) | 75(93.8) | — | 0.6(0.108,3.346) | 0.625 |
| Xerophthalmia | 20(100.0) | 79(98.8) | 0.253 | Not estimable | 0.615 |
| Raynaud's phenomenon | 4(20.0) | 14(17.5) | — | 1.179(0.342,4.065) | 0.753 |
| Rampant caries | 8(40.0) | 29(36.3) | 0.97 | 1.172(0.43,3.2) | 0.756 |
| Purpuric rash | 0(0.0) | 5(6.3) | — | Not estimable | 0.58 |
| Fatigue | 11(55.0) | 39(48.8) | 0.25 | 1.285(0.48,3.437) | 0.617 |
| Cough | 7(35.0) | 17(21.3) | — | 1.995(0.689,5.78) | 0.243 |
| Dyspnea | 6(30.0) | 10(12.5) | — | 3(0.937,9.603) | 0.084 |
| Lymphadenopathy | 5(25.0) | 16(20.0) | — | 1.333(0.422,4.215) | 0.759 |
| Arthritis | 2(10.0) | 9(11.3) | — | 0.877(0.174,4.417) | 1 |
| Parotid enlargement | 2(10.0) | 5(6.3) | — | 1.667(0.299,9.295) | 0.625 |
| Splenomegaly | 1(5.0) | 8(10.0) | — | 0.474(0.056,4.024) | 0.683 |
| Interstitial lung disease | 9(45.0) | 24(30.0) | 1.628 | 1.909(0.701,5.202) | 0.202 |
| WBC, ×109/L | 4.84(4.09,5.77) | 4.78(3.78,6.08) | — | — | 0.599 |
| NEUT, ×109/L | 2.53(2.16,3.41) | 2.85(2.02,3.82) | — | — | 0.485 |
| LYMPH, ×109/L | 1.62(1.30,1.98) | 1.35(1.11,1.70) | — | — | 0.064 |
| RBC, ×109/L | 4.16 ± 0.43 | 3.90 ± 0.64 | — | — | 0.079 |
| HB, g/L | 130.00(119.50,135.50) | 121.00(108.00,130.75) | — | — | 0.015 |
| PLT, ×109/L | 191.50(164.00,210.00) | 169.00(127.00,212.75) | — | — | 0.135 |
| ALT, IU/L | 24.50(18.00,41.25) | 18.00(12.25,33.00) | — | — | 0.081 |
| AST, IU/L | 24.50(19.25,41.00) | 23.00(18.25,29.75) | — | — | 0.209 |
| TBIL, μmol/L | 11.82(10.08,15.56) | 10.70(8.26,14.34) | — | — | 0.708 |
| ALB, g/L | 40.00(39.05,42.75) | 40.00(38.00,42.93) | — | — | 0.679 |
| GGT, IU/L | 24.00(19.00,43.25) | 27.50(16.25,80.50) | — | — | 0.829 |
| LDH, IU/L | 200.50(159.00,237.00) | 186.00(157.50,220.00) | — | — | 0.535 |
| CK, IU/L | 88.50(45.25,103.50) | 53.50(40.25,81.75) | — | — | 0.021 |
| BUN, mmol/L | 4.56(4.14,5.37) | 4.81(3.78,6.26) | — | — | 0.663 |
| Cr, μmol/L | 58.95(55.20,65.20) | 61.50(52.68,69.75) | — | — | 0.477 |
| K, mmol/L | 3.96 ± 0.25 | 3.99 ± 0.38 | — | — | 0.802 |
| Cl, mmol/L | 106.50(104.50,109.00) | 106.00(105.00,108.00) | — | — | 0.852 |
| eGFR, ml/min/1.73m2 | 94.11(87.41,99.67) | 90.63(76.62,101.52) | — | — | 0.379 |
| D-Dimer, mg/L | 0.33(0.25,0.53) | 0.49(0.29,1.12) | — | — | 0.057 |
| Hypergammaglobulinemia | 4(20.0) | 43(53.8) | 7.316 | 0.215(0.066,0.7) | 0.007 |
| IgG, g/L | 13.25 ± 3.11 | 14.10(11.35,19.13) | — | — | 0.192 |
| Hyper-IgG (> 16.2 g/L) | 2(10.0) | 29(36.3) | 5.154 | 0.195(0.042,0.903) | 0.023 |
| IgA, g/L | 2.31(1.75,3.37) | 3.03(2.14,3.80) | — | — | 0.173 |
| Hyper-IgA (> 3.78 g/L) | 2(10.0) | 21(26.3) | — | 0.312(0.067,1.461) | 0.148 |
| IgM, g/L | 1.15(0.73,1.70) | 1.28(0.80,2.09) | — | — | 0.282 |
| Hyper-IgM (> 2.63 g/L) | 1(5.0) | 12(15.0) | — | 0.298(0.036,2.441) | 0.456 |
| Hypocomplementemic | 5(25.0) | 43(53.8) | 5.298 | 0.287(0.095,0.865) | 0.021 |
| C3, g/L | 0.82(0.76,0.95) | 0.77(0.69,0.92) | — | — | 0.281 |
| Low C3 (＜ 0.7 g/L) | 2(10.0) | 26(32.5) | 4.018 | 0.231(0.05,1.07) | 0.045 |
| C4, g/L | 0.19(0.17,0.22) | 0.17(0.14,0.20) | — | — | 0.02 |
| Low C4 (＜ 0.16 g/L) | 4(20.0) | 34(42.5) | 3.438 | 0.338(0.104,1.103) | 0.064 |
| ANA titres ≥ 1:320 | 7(35.0) | 34(42.5) | 0.372 | 0.729(0.263,2.021) | 0.542 |
| CRP, mg/dL | 0.31(0.25,0.59) | 0.27(0.17,0.51) | — | — | 0.673 |
| Elevated CRP (＞ 8 mg/L) | 2(10.0) | 13(16.3) | — | 0.573(0.118,2.772) | 0.729 |
| ESR, mm/h | 10.00(5.50,15.50) | 16.00(11.00,36.50) | — | — | 0.006 |
| Elevated ESR (＞ 20 mm/h) | 4(20.0) | 37(46.3) | 4.558 | 0.291(0.089,0.946) | 0.033 |
| ESSDAI scores | 8.50(2.50,12.00) | 6.00(3.00,15.00) | — | — | 0.799 |
| Positive LSGB | 20/20(100) | 47/80(97.9) | — | 0.979(0.94,1.02) | 1.000 |
| Focal lymphocytic infiltration count | 3(2,3.5) | 4(2,5) | — | — | 0.137 |

WBC, white blood cell; NEUT, neutrophil; LYMPH, lymphocyte; RBC, red blood cell; Hb, hemoglobin; PLT, platelet; ALT, alanine transaminase; AST, aspartate aminotransferase; TBIL, total bilirubin; ALB, albumin; GGT, gamma glutamyl transferase; LDH, lactate dehydrogenase; CK, creatine kinase; BUN, blood urea nitrogen; Cr, serum creatinine; eGFR, estimated glomerular filtration rate; Ig, immunoglobulin; C3, complement 3; C4, complement 4; RF, rheumatoid factor; ESR, erythrocyte sedimentation rate; CRP, C-reactive protein; ANA, antinuclear antibodies; ESSDAI, EULAR Sjögren’s syndrome disease activity index; AMA-M2, anti-mitochondrial antibody M2; ACL, anti-cardiolipin; LSGB, Labial salivary gland biopsy.

Not estimable means ORs were not reported for variables with zero cells or near-complete separation.

**Supplementary Table 3. | Incidence of coexisting autoimmune diseases during follow-up in primary Sjögren’s disease patients with and without anti-CENP-B antibody**

| Supplementary Table 3. Incidence of coexisting autoimmune diseases during follow-up in primary Sjögren’s disease patients with and without anti-CENP-B antibody | | |
| --- | --- | --- |
|  | Anti-CENP-B antibody positivity, n = 97 | Anti-CENP-B antibody negative, n = 1038 |
| SLE | 1 | 9 |
| RA | 2 | 15 |
| DM | 1 | 2 |
| ASS | 0 | 4 |
| Systemic vasculitis | 0 | 3 |
| APS | 0 | 3 |
| AOSD | 0 | 1 |
| PBC | 0 | 6 |
| AIH | 0 | 2 |
| PMR | 0 | 2 |

SLE, Systemic lupus erythematosus; RA, Rheumatoid Arthritis; DM, dermatomyositis;  ASS, antisynthetase syndrome; APS, antiphospholipid syndrome; AOSD, Adult onset still’s disease; PBC, primary biliary cholangitis;AIH, autoimmune hepatitis; PMR, Polymyalgia Rheumatica

## 2 Supplementary Figures

**Supplementary Figure 1. Kaplan-Meier curves**

**
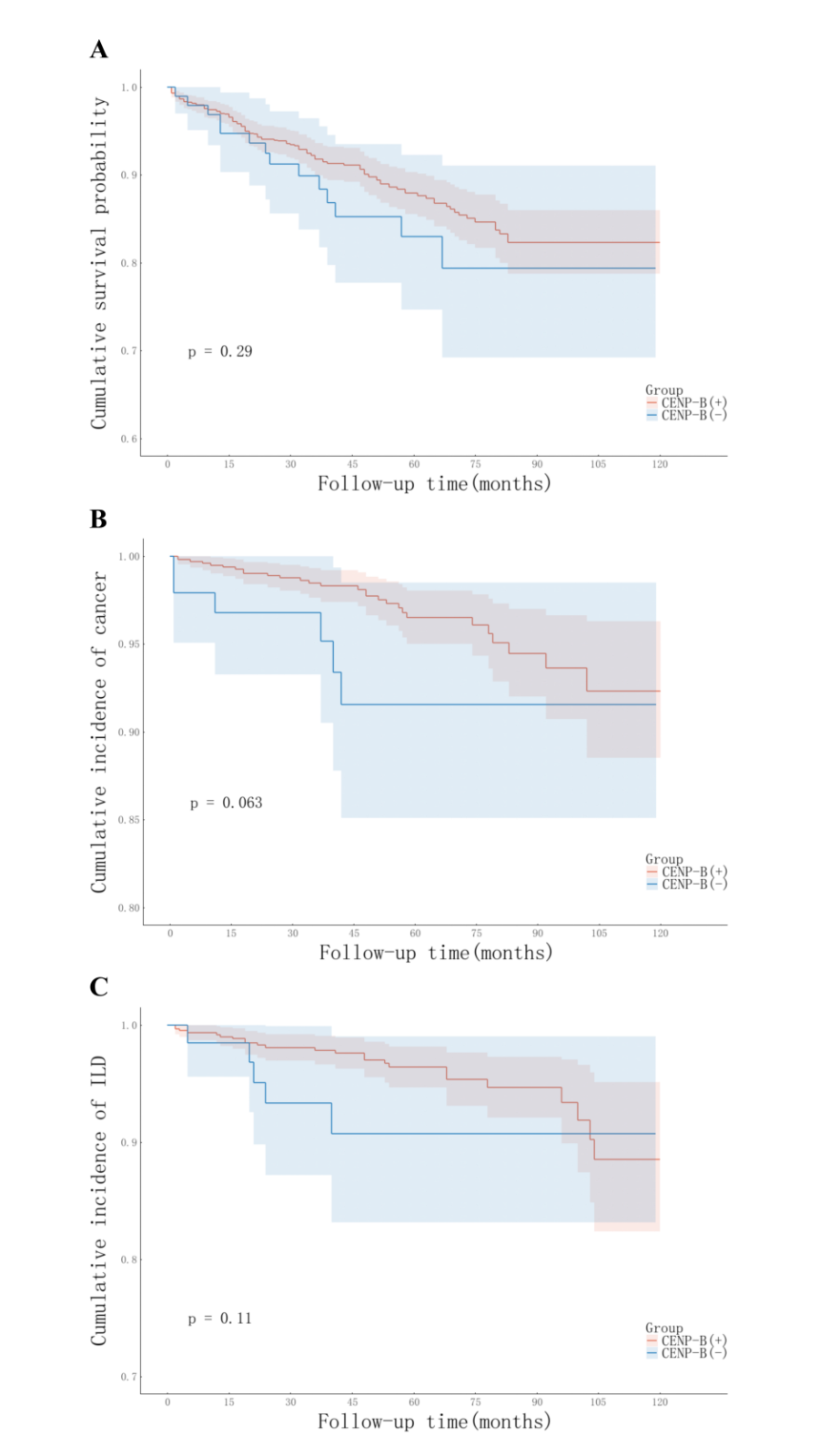
**

**Supplementary Figure 1.** Kaplan-Meier curves. (A) Kaplan‒Meier curves for all-cause death in patients with pSD stratified by anti-CENP-B antibody. (B) Kaplan‒Meier curves for cancer patients with pSD stratified by anti-CENP-B antibody. (B) Kaplan‒Meier curves for ILD patients with pSD stratified by anti-CENP-B antibody.
